# Epigenetic and Liquid Biopsy Biomarkers in Prostate Cancer: Bridging Tumor Heterogeneity and Clinical Implementation

**DOI:** 10.3390/cancers18030389

**Published:** 2026-01-27

**Authors:** Joanna Robaczyńska, Maciej Maj, Adam Kiljańczyk, Bartosz Pastuszek, Emilia Reducha, Aleksandra Nurkiewicz, Milena Kiljańczyk

**Affiliations:** 1 Department of Genetics and Pathology, International Hereditary Cancer Center, Pomeranian Medical University, ul. Unii Lubelskiej 1, 71-252 Szczecin, Poland; s089400@student.wum.edu.pl (J.R.); adam.kiljanczyk@pum.edu.pl (A.K.); 2 Department of Histology and Embryology, Medical University of Warsaw, 02-004 Warsaw, Poland; s085179@student.wum.edu.pl; 3 Read-Gene, ul. Alabastrowa 8, Grzepnica, 72-003 Dobra, Poland; 4 Department of Diagnostic Imaging and Interventional Radiology, Pomeranian Medical University Hospital No. 1, 71-252 Szczecin, Poland; 5 Faculty of Medicine, Medical University of Warsaw, 02-091 Warsaw, Poland; s088362@student.wum.edu.pl (B.P.); s088407@student.wum.edu.pl (E.R.); s088340@student.wum.edu.pl (A.N.)

**Keywords:** prostate cancer, liquid biopsy, ctDNA, non-coding RNA, biomarkers, precision oncology, epigenetics

## Abstract

Prostate cancer (PCa) is the most common malignancy in men, characterized by significant genetic and epigenetic heterogeneity, which complicates both diagnosis and treatment processes. Epigenetic mechanisms—including DNA methylation, chromatin remodeling, and dysregulated non-coding RNAs (miRNAs, lncRNAs, circRNAs)—contribute to tumor initiation, progression, and therapy resistance, offering promising diagnostic and prognostic biomarker opportunities. Liquid biopsy technologies, such as circulating tumor DNA (ctDNA), circulating tumor cells (CTCs), and exosomes, allow minimally invasive, real-time monitoring of tumor evolution and resistance mechanisms, complementing traditional biomarkers like PSA and supporting precision oncology approaches. Clinically implemented assays, including PCA3, ConfirmMDx, and ExoDx Prostate, along with emerging multi-analyte panels, enhance risk stratification, reduce unnecessary biopsies, and guide therapeutic decisions. Integration of epigenetic and liquid biopsy biomarkers into multimodal diagnostic pathways has the potential to support personalized management of prostate cancer; however, many still require further validation and optimization.

## Simple Summary

Prostate cancer is one of the most common cancers in men and is characterized by substantial intertumoral heterogeneity, which makes diagnosis and treatment challenging. Conventional tests, such as prostate-specific antigen measurement, do not always reliably identify aggressive disease or predict treatment response. Recent research indicates that epigenetic alterations and tumor-derived material detected in blood or urine can provide more accurate information about tumor biology. This review summarizes current knowledge on epigenetic biomarkers and liquid biopsy approaches, including analyses based on DNA, RNA, and circulating tumor cells. The main intention of this review is to present the current status of various biomarkers and to assess whether they are, or have the potential to be, routinely used in clinical practice based on current data and guidelines. We discuss how these tools may help reduce unnecessary biopsies, improve risk stratification, and support more personalized treatment decisions. Integrating these biomarkers into clinical practice in the future could improve prostate cancer management and patient outcomes; however, many still require further validation and optimization, for example, by combining multiple biomarkers with other clinical data.

## 1. Introduction

Prostate cancer (PCa) is the most prevalent malignancy in men, accounting for approximately 20% of all detected tumors worldwide [1]. Increasing evidence indicates that an individual’s genetic profile plays a crucial role in prostate cancer susceptibility and clinical course. Beyond inherited and somatic genetic alterations, epigenetic mechanisms play a central role in prostate cancer initiation, progression, therapeutic resistance, and clonal evolution. Aberrant DNA methylation altered chromatin remodeling, and dysregulated expression of non-coding RNAs, including microRNAs (miRNAs) and long non-coding RNAs (lncRNAs), contribute to tumor plasticity and disease progression [2,3].

Despite substantial advances in molecular characterization, effective clinical management of prostate cancer remains challenging due to pronounced inter- and intratumoral heterogeneity. Prostate tumors are frequently multifocal and multiclonal [2], with genetically distinct subclones coexisting within the same patient. This biological complexity complicates precision oncology, promotes resistance to systemic therapies, and limits the predictive value of currently used biomarkers. Consequently, established diagnostic tools such as prostate-specific antigen (PSA), Prostate Health Index (PHI), Prostate Cancer Antigen 3 (PCA3, also referred to as DD3), and the Prostate-Specific Kallikrein, 4Kscore, while clinically useful, often lack sufficient specificity and may not fully capture disease heterogeneity [4].

To address these limitations, current research increasingly focuses on the development of more precise molecular biomarkers. Promising candidates include nucleic acid-based markers, such as microRNAs (miRNAs), long non-coding RNAs (lncRNAs), circular RNAs (circRNAs), and DNA-derived signatures, which offer deeper insights into tumor biology. In parallel, liquid biopsy approaches—analyzing circulating tumor-derived biomarkers in blood or urine—have emerged as valuable tools to improve PSA specificity, reduce unnecessary tissue biopsies, and enable dynamic monitoring of tumor evolution over time [5].

Precision oncology in prostate cancer enables treatment individualization and supports ongoing assessment of molecular tumor dynamics through repeated tissue or liquid biopsy sampling [6]. This approach is particularly relevant given the limitations of current therapeutic strategies, including treatment-related toxicity, limited long-term efficacy, and frequent development of resistance. Consequently, several innovative treatment strategies are under investigation, including nanotechnology-based therapies, gene therapy, phytochemicals with anticancer properties, and immunotherapeutic approaches targeting the tumor microenvironment [7].

This review provides a comprehensive overview of emerging diagnostic and therapeutic strategies in prostate cancer, extensively studied in the recent years. Its primary aim is to present the current status of various biomarkers and to assess whether they are, or have the potential to be, routinely implemented in clinical practice based on current data and guidelines. We highlight areas where the evidence supports clinical application, discuss key limitations, and outline future directions to enhance the personalized management of prostate cancer.

## 2. Materials and Methods

For this narrative review, records were identified through PubMed/MEDLINE (last 15 years, 2011–2025, were screened) using the following search terms: prostate cancer, liquid biopsy, non-coding RNA, miRNA, exosomes, ctDNA, CTC, DNA repair pathways, DNA modification, histone methylation, chromatin remodeling, targeted therapy, and personalized therapy. Records excluded were letters to the editor, editorials, research protocols, case reports, brief correspondence, non-English articles, animal studies, studies involving multiple cancer types, and studies with fewer than 20 patients. Additional records were identified through reference screening of included articles.

Following database searching, a total of 335 records met the initial eligibility criteria and were subjected to a stepwise selection process, including title and abstract screening followed by full-text assessment. During manuscript preparation, an additional three relevant articles were identified and included based on reference tracking and content relevance. Study selection prioritized peer-reviewed journals with established editorial standards and relevance to clinical or translational prostate cancer research. Particular emphasis was placed on studies demonstrating practical or potential clinical applicability, especially those addressing diagnostic, prognostic, or therapeutic implications. Articles of borderline relevance or methodological uncertainty were discussed among members of the research team prior to final inclusion. Ultimately, a total of 134 studies were included in the final narrative synthesis.

## 3. Epigenetic Biomarkers in Prostate Cancer

Epigenetic alterations play a pivotal role in prostate tumorigenesis and offer promising biomarkers for detection and risk stratification. DNA methylation changes are among the earliest and most stable epigenetic events in PCa. For example, *GSTP1* (glutathione S-transferase P1) is hypermethylated and silenced in ~90% of PCa, a hallmark not seen in normal prostate tissue. Hypermethylation of other tumor suppressors (e.g., *APC*, *RASSF1*, *RARB*) is also frequent, and these changes can be detected noninvasively. A study of 514 patients found ≥80% of urine samples from PCa patients harbored methylation of *GSTP1*, *RASSF1* or *RARB*, highlighting the value of cell-free DNA (cfDNA) methylation in urine [8]. In plasma, emerging assays capture methylated cfDNA to distinguish cancer; for instance, genome-wide cfDNA methylome analysis can differentiate PCa from benign disease and even predict outcomes [8,9].

Clinically, the ConfirmMDx tissue test leverages an epigenetic field effect by PCR-detecting *GSTP1*, *APC*, and *RASSF1* methylation in histologically negative biopsies [10]. This test, included in NCCN and EAU guidelines, helps identify men at risk of occult cancer after a negative biopsy (sensitivity ~74%, specificity ~60% [11]. Chromatin remodeling and histone modifications are also perturbed in PCa. Overexpression of EZH2 (catalyzing H3K27 trimethylation) and global loss of certain histone marks (e.g., H3K27me3) have been associated with aggressive disease [12]. While histone marks are harder to measure clinically, research shows distinctive patterns (e.g., reduced H3K27me3) correlate with higher Gleason grade and progression [13]. Alterations in chromatin modifiers (e.g., CHD1, ARID1A mutations) further underscore the role of chromatin structure in PCa; these changes may become biomarkers or therapeutic targets as our understanding grows [12]. Table 1 provides a summary of the main methylated genes detected as biomarkers, including their current clinical utility, research versus clinical status, and noted limitations.

Another major epigenetic layer involves non-coding RNAs, notably microRNAs and long non-coding RNAs, which regulate gene expression post-transcriptionally. These molecules are dysregulated in PCa and can be detected in tissues and liquid biopsy samples.

### 3.1. MicroRNAs (miRNAs)

MiRNAs are small non-coding RNAs that regulate gene expression at the post-transcriptional level, thereby directly influencing protein levels in human cells. Due to their regulatory role, miRNAs have been extensively investigated as potential biomarkers in PCa. Numerous studies have demonstrated that specific miRNAs are either upregulated or downregulated in this disorder, and that their expression profiles may correlate with disease progression and therapeutic response.

Importantly, miRNA signatures may complement established diagnostic tools such as PSA testing and prostate volume assessment, particularly in patients classified within the diagnostic “grey zone” [28]. For example, increased expression of miR-145 has been observed in patients who respond favorably to neoadjuvant radiotherapy, defined by a PSA increase of less than 2.0 ng/mL per year [29].

In addition, urinary miRNAs such as miR-148a and miR-375 have been identified as biomarkers for PCa [30]. Their clinical relevance lies in two major aspects: first, they enhance the diagnostic accuracy of PSA testing, and second, they enable more precise clinical decision-making for patients within the grey diagnostic zone.

Several studies have also focused on distinguishing benign prostatic hyperplasia (BPH) from PCa based on miRNA expression profiles. Analyses revealed significant downregulation of miR-221-5p and miR-708-3p in PCa compared with BPH, suggesting their potential utility as discriminatory biomarkers [31].

Furthermore, differences in miRNA expression have been further reported between metastatic Castration-Resistant Prostate Cancer (mCRPC) and localized PCa. In this context, 63 miRNAs were found to be upregulated and four downregulated in mCRPC. Notable examples include miR-151-3p, miR-423-3p, miR-126, miR-152, miR-21, and miR-16 [32].

Additional miRNAs, such as miR-205 and miR-214, as well as miR-221 and miR-99b, have shown significant downregulation in PCa patients [33]. However, for some of these miRNAs, a clear correlation with clinical parameters has not yet been established, indicating the need for further research and validation.

Moreover, certain miRNAs have also demonstrated other important clinical features. For instance, miR-200c has been shown to improve the predictive accuracy of the Gleason score, while miR-200b expression is associated with bone metastasis, bilateral tumor involvement, and PSA levels exceeding 10.0 ng/mL [34]. Finally, the entire miR-200 family has been linked to disease progression and overall survival in CRPC [35].

Most miRNA biomarkers are still at the research stage and currently lack sufficient evidence to outperform existing diagnostic approaches. It is unlikely that a single miRNA–based test will be clinically useful on its own; rather, a combined approach integrating multiple miRNAs and other clinical parameters is expected to be more effective. Some preliminary efforts in this direction have already been made. For example, one commercial test analyzes a panel of urinary exosome miRNAs. However, it is not included in mainstream clinical guidelines, and to our information, the manufacturer does not disclose which specific miRNAs are detected by the assay [36].

Beyond their value as diagnostic and prognostic biomarkers, miRNAs are also being actively explored as therapeutic agents in prostate cancer. A recent study demonstrated ligand-directed delivery of chemically modified miRNAs selectively to prostate cancer cells, significantly improving cellular uptake, endosomal escape, and functional gene silencing efficacy. Importantly, this approach leveraged small-molecule targeting to enhance tumor specificity, directly addressing key translational barriers such as off-target effects and inefficient intracellular delivery. These findings highlight how biomarker-guided targeting strategies can enable epigenetic modulation at the tumor-cell level, offering a concrete example of how miRNA biology may be translated into clinically actionable precision therapeutics in prostate cancer [37]. Table 2 summarizes the key miRNAs identified as biomarkers, highlighting their clinical relevance, stage of research/clinical implementation, and associated limitations.

### 3.2. Long Non-Coding RNAs (lncRNAs)

Long non-coding RNAs (lncRNAs) play key regulatory roles in chromatin remodeling, transcriptional control, and cell signaling, and are increasingly recognized as promising biomarkers in PCa. Recent evidence indicates that the tumor microenvironment itself contributes to lncRNA dysregulation: a comparative analysis of cancer-associated fibroblasts and benign prostatic hyperplasia-derived fibroblasts identified 17 differentially expressed lncRNAs, 15 of which demonstrated potential diagnostic relevance, underscoring the importance of stromal–epithelial interactions in PCa biology [63].

The most clinically established lncRNA is PCA3, which is overexpressed approximately 100-fold in more than 90% of prostate cancers and is virtually undetectable in non-prostatic tissues. PCA3 is secreted into urine and forms the basis of the FDA-approved Progensa PCA3 assay, one of the earliest urine-based molecular diagnostics in oncology [64]. Although PCA3 exhibits only moderate sensitivity as a standalone marker, it provides information independent of PSA and is particularly useful in guiding repeat biopsy decisions in men with prior negative biopsies [65].

Beyond PCA3, several oncogenic lncRNAs have emerged as candidate biomarkers of aggressive disease. SChLAP1, which antagonizes the SWI/SNF chromatin remodeling complex, is overexpressed in approximately 25% of high-grade tumors and is associated with metastasis and poor clinical outcomes [66]. While not yet implemented in routine clinical practice, SChLAP1 and other lncRNAs, including members of the PCAT family and MALAT1, show promise as tissue- or urine-based biomarkers for risk stratification and prognosis [67,68].

More recently, lncRNAs linked to cuproptosis—a copper-dependent form of regulated cell death driven by mitochondrial dysfunction—have been described in PCa. A panel including AC005790.1, AC011472.4, AC099791.2, AC144450.1, LIPE-AS1, and STPG3-AS1 demonstrated both diagnostic and prognostic value, highlighting a novel mechanistic axis with potential translational relevance [69]. Table 3. presents an overview of the main lncRNAs proposed as biomarkers, detailing their clinical significance, whether they are still in research or used clinically, and their limitations.

## 4. Circular RNAs (circRNAs)

Circular RNAs (circRNAs) are a relatively recently characterized class of endogenous RNAs generated through back-splicing [81]. In prostate cancer (PCa), aberrant circRNA expression has been consistently reported across primary studies, and a 2022 systematic review and meta-analysis concluded that circRNAs demonstrate moderate overall diagnostic accuracy, supporting their potential as diagnostic biomarkers [81]. Early functional investigations further suggest that specific circRNAs may reflect biological aggressiveness; for example, circSLC39A10 has been shown to promote PCa progression via a miR-936/PROX1/β-catenin (Wnt) signaling axis and has been discussed as a candidate biomarker in this context [82].

CircRNAs, including tumor-associated circRNAs, have also been explored in minimally invasive settings such as serum-based studies in men undergoing prostate biopsy; however, findings to date remain inconsistent, and several proposed circRNA candidates have failed to reliably discriminate prostate cancer from biopsy-negative controls [83]. While tissue-based profiling and liquid biopsy approaches hold promise for the identification of circRNA-based diagnostic and progression biomarkers in PCa, substantial challenges remain. In particular, the field requires standardized analytical assays and large, independent prospective validation cohorts before any circRNA can be considered clinically actionable [81,84].

## 5. Liquid Biopsy

In the last couple of years, significant progress has been made in the diagnosis and monitoring of patients with PCa. As it became clear that PCa is not a homogeneous disease, the assessment of its molecular characteristics has proven highly beneficial. A variety of molecules have shown clinical utility, including non-coding RNAs (ncRNAs), circulating tumor DNA (ctDNA), exosomes, and numerous metabolomic biomarkers [85].

**Figure 1:**
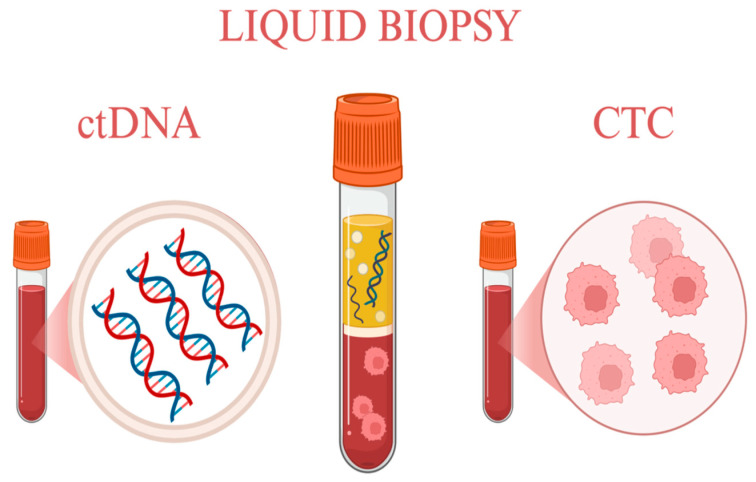
Most common liquid biopsy methods. Created in https://BioRender.com.

The analysis of these biomarkers has enabled the development of a novel therapeutic approach for men affected by this disease—namely, the evaluation of tumor-derived markers in blood and urine samples through liquid biopsy. This method is particularly valuable for detecting mechanisms of resistance to the most common treatment, androgen receptor inhibitors. PCa frequently progresses to mCRPC, where such an approach offers new therapeutic opportunities [86]. These concepts are illustrated in Figure 1, which provides a schematic overview of the most common liquid biopsy methods used in prostate cancer.

### 5.1. Circulating Tumor DNA (ctDNA)

As previously mentioned, liquid biopsy has gained increasing attention in the management of PCa due to its non-invasiveness and its ability to monitor tumor dynamics in real time. One of the most widely implemented approaches is based on circulating tumor DNA (ctDNA) analysis [87]. ctDNA enables the assessment of tumor heterogeneity and clonal evolution with greater precision than single-site tissue biopsies or conventional biomarkers such as PSA [88]. This is particularly important given the tendency of PCa to undergo treatment-driven progression [89].

Comprehensive ctDNA profiling provides information on somatic mutations, copy number alterations, and structural rearrangements (e.g., in *AR* or *MYC*), as well as disruptions in DNA damage repair genes, including *BRCA1/2*. Importantly, it also enables the detection of treatment resistance mechanisms. This aspect is of clinical relevance, as ctDNA facilitates the monitoring of resistance to androgen receptor inhibitors, allowing the identification of alterations—such as gene amplifications or activating mutations—that drive therapeutic refractoriness [90,91]. As a result, responders and non-responders can be distinguished early, preventing prolonged use of ineffective therapies and enabling timely adjustment of treatment strategies [92].

In patients with mCRPC, ctDNA has emerged as one of the most important prognostic and predictive biomarkers. It supports the identification of subgroups likely to benefit from PARP inhibitors (e.g., carriers of pathogenic *BRCA1/2* variants) and allows for more accurate monitoring of global molecular tumor burden than standard radiologic methods. Notably, patients with ctDNA-positive mCRPC demonstrate worse survival outcomes compared with those who are ctDNA-negative [91].

From a clinical perspective, ctDNA analysis also offers several practical advantages. It may be cost-effective in the long term, reduces reliance on difficult-to-obtain bone tissue (a frequent metastatic site in mCRPC), and generally exhibits higher analyte abundance than circulating tumor cells (CTCs) in early-stage disease [93]. Nevertheless, the two approaches are complementary: certain biomarkers—particularly AR-V7 and some other indicators of resistance to androgen receptor signaling inhibitors—are more reliably detected in CTCs [94] (Table 4).

Several limitations of ctDNA analysis should also be acknowledged. Clonal hematopoiesis may lead to false-positive variant calls; therefore, the use of matched white blood cell DNA or strict adherence to validated protocols is recommended [95]. Additionally, in cases of minimal residual disease or in tumors with predominantly osseous metastases, the circulating tumor fraction may be insufficient for reliable ctDNA detection [96].

In summary, although ctDNA analysis is not without limitations, it represents one of the most powerful and promising tools currently available for precision monitoring and therapeutic guidance in prostate cancer.

### 5.2. Circulating Tumor Cells (CTCs)

As previously mentioned, another liquid biopsy method is based on CTCs. CTCs are FDA-approved predictive biomarkers in metastatic hormone-sensitive prostate cancer (mHSPC) as well as mCRPC. Their assessment contributes to improved patient management and enhances current prognostic tools.

The CTC count is highly predictive of overall survival (OS), progression-free survival (PFS), and 7-month PSA response. It enables patient stratification before therapy selection, as a high CTC count (>5) predicts poorer OS and PFS [97,98,99].

Moreover, CTC levels correlate with both biological and clinical characteristics of the tumor. Higher CTC counts are associated with increased mutational burden and greater genomic complexity [98]. Additionally, CTC counts have been shown to correlate with white blood cell count (WCC) and platelet cloaking [100].

Because CTCs are an important diagnostic tool, but traditional detection methods were limited to identifying only epithelial/EMT-like CTCs (EpCAM-based), newer approaches have been developed. Single-cell and transcriptomic technologies have expanded the detectable range of CTC subtypes and improved their characterization [101,102].

### 5.3. Extracellular Vesicles and Exosomes

Exosomes are ultrastructural spherical vesicles enclosed by a lipid bilayer and released as part of the cellular secretory pathway. As products of cellular secretion, they carry a diverse molecular cargo, including nucleic acids, proteins, and lipids. Among their protein components, proteasome subunit alpha type-6 (PSM-E) has recently been proposed as a novel potential biomarker [103].

Of particular importance are exosomal nucleic acids, especially microRNAs and long non-coding RNAs (lncRNAs), which show strong potential as novel biomarkers for PCa [104]. In addition to proteins and lipids, exosomes function within a broader liquid biopsy landscape, interacting with and complementing other circulating components such as CTCs in mediating tumor progression and intercellular communication.

**Figure 2:**
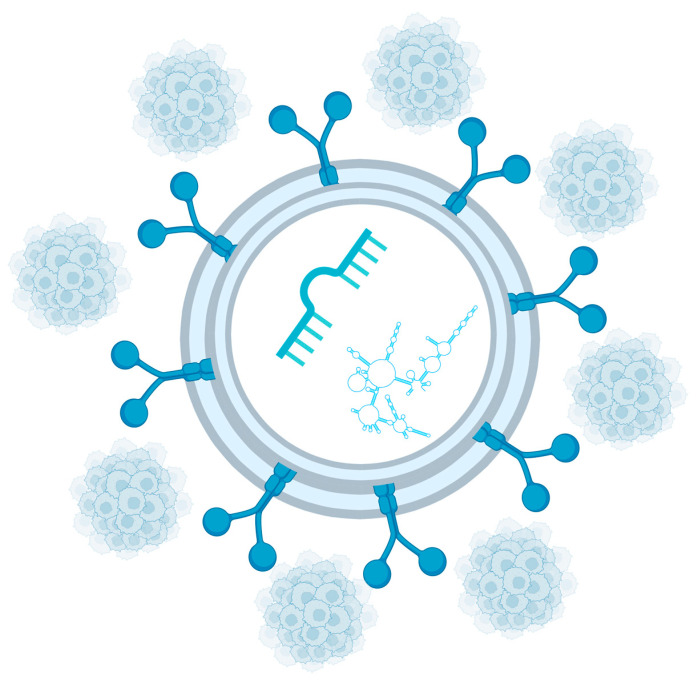
Important exosome cargo includes miRNAs and other non-coding RNAs. Circulating tumor cells are another key component of liquid biopsy. Created in https://BioRender.com.

By transporting bioactive molecules, exosomes facilitate tumor dissemination and contribute to disease progression, which is particularly relevant in metastatic PCa. Consequently, exosomal profiling—especially when integrated with CTC analysis—can significantly improve the quality of patient care and offers new opportunities for the development of advanced diagnostic and therapeutic strategies [105]. These relationships are illustrated in Figure 2, which depicts exosomes as carriers of miRNAs and other non-coding RNAs and highlights their interplay with CTCs within the liquid biopsy-driven PCa evaluation.

Taken together, miRNAs offer a new approach in scenarios where conventional biomarkers and imaging provide insufficient information. An additional insight into tumor aggressiveness, treatment response, and disease progression supports further clinical decisions, including biopsy triage, treatment escalation, and early identification of systemic disease.

## 6. Critical Technical and Translational Limitations

Despite rapid technological advances and the growing number of proposed epigenetic and liquid biopsy-based biomarkers, their widespread clinical implementation remains constrained. These constraints reflect fundamental biological, analytical, and organizational barriers. Recent high-impact reviews emphasize that these limitations are not solely attributable to insufficient clinical validation. Instead, they reflect intrinsic properties of the biological material, tumor heterogeneity, and current technological constraints of available assays [106,107,108].

### 6.1. CTCs Are Not Suitable for Screening

CTCs occur at extremely low frequencies in peripheral blood and exhibit short survival times. Their detection depends on sufficiently high tumor burden and active cellular shedding from primary or metastatic lesions. Consequently, CTC-based assays show very limited sensitivity in early-stage disease and are not suitable for population screening or early cancer detection. This has been consistently shown in multiple reviews [106,109]. Their clinical utility is therefore largely restricted to advanced disease, where they serve as prognostic or predictive biomarkers.

### 6.2. ctDNA: Low Tumor Fraction and Clonal Hematopoiesis Interference

ctDNA typically represents only a small fraction of total circulating cfDNA, particularly in early-stage disease or after curative-intent treatment, substantially limiting analytical sensitivity. An additional major challenge is clonal hematopoiesis of indeterminate potential (CHIP), whereby somatic mutations originating from hematopoietic cells may be misclassified as tumor-derived alterations. Reviews in *NEJM* and *Nature Reviews Clinical Oncology* show that these factors significantly restrict the use of ctDNA for population screening. They also necessitate rigorous biological and bioinformatic controls for accurate interpretation [107,108,110,111].

### 6.3. miRNA: Hemolysis Sensitivity and Lack of Normalization Consensus

Circulating miRNA analysis is particularly vulnerable to pre-analytical variability. Hemolysis during sample collection or processing releases miRNAs from erythrocytes and platelets, profoundly altering expression profiles. This issue is compounded by the lack of a universally accepted normalization strategy. miR-16, which is frequently used as a reference control, is itself highly sensitive to hemolysis. This limits reproducibility and cross-study comparability [106,112].

### 6.4. PCA3: Normalization to PSA mRNA Rather than Creatinine

The PCA3 urine test is based on the ratio of PCA3 to PSA mRNA, which enhances prostate specificity but simultaneously renders the result dependent on the amount of prostate-derived RNA and sampling conditions, such as the effectiveness of DRE. The absence of normalization to urinary creatinine means that urine dilution is not directly controlled, potentially affecting inter-patient and inter-center comparability [113,114].

### 6.5. Tumor Heterogeneity Versus Increasing Analytical Sensitivity

Tumors exhibit pronounced spatial and clonal heterogeneity. Increasing analytical sensitivity, such as with deep NGS, enables the detection of rare variants. However, it also amplifies biological noise and increases the risk of overrepresenting individual subclones, which complicates clinical decision-making [107,111].

### 6.6. Cost and Complexity of Multimarker Panels

Multimarker genomic and epigenetic panels may partially address tumor heterogeneity but are associated with high costs, complex bioinformatic workflows, and challenges related to clinical validation and reimbursement. Contemporary reviews indicate that, despite promising translational results, scalability and routine clinical use of such panels remain limited [89,108].

### 6.7. Clinical Bottlenecks: Pre-Analytical Variability, SOPs, and Logistics

A major translational barrier for liquid biopsy implementation is the lack of standardized procedures for sample collection, processing, and storage. Variations in blood collection tubes, time to plasma separation, transport conditions, and freeze–thaw cycles can introduce variability. In some cases, this variability exceeds the biological signal itself. Both *Nature*-group reviews and *NEJM* commentaries emphasize that without harmonized SOPs and stringent quality control, liquid biopsy results remain difficult to compare across centers and studies [106,107].

### 6.8. Perspective of Clinical Guidelines (ESMO, NCCN)

The cautious stance of contemporary clinical guidelines further reflects the technical and biological limitations outlined above. Both the European Society for Medical Oncology (ESMO) and the National Comprehensive Cancer Network (NCCN) acknowledge the potential value of selected molecular assays in specific clinical contexts; however, they do not recommend routine clinical use of liquid biopsy-based biomarkers for population screening or as part of the standard diagnostic pathway for prostate cancer.

These guidelines emphasize that the available evidence is often heterogeneous and frequently derived from retrospective studies or selected cohorts. Moreover, the impact of many molecular assays on hard clinical endpoints, such as cancer-specific survival, remains insufficiently demonstrated. Consequently, ctDNA, CTCs, and RNA—based panels are currently considered primarily within clinical trials, advanced disease monitoring, or selected decision-making scenarios rather than routine clinical use in primary diagnostics [115,116].

The major technical and translational limitations discussed above are summarized in Table 5.

## 7. Practical Clinical Use of Molecular and Epigenetic Biomarkers

Urinary biomarkers have become an important component in the detection and risk stratification of clinically significant prostate cancer (csPC). Among the most studied markers is PCA3, an FDA-approved non-coding RNA assay primarily used to guide repeat biopsy decisions in men with prior negative biopsies. PCA3 offers higher specificity than PSA and is independent of age, prostate volume, or inflammation, though its role in predicting tumor aggressiveness and progression remains limited [52]. Another extensively studied urinary biomarker is the TMPRSS2–ERG gene fusion, absent in benign prostate tissue and detectable in approximately 49% of European men. When combined with PSA testing and PCA3 in commercial assays such as Mi-Prostate Score/MyProstateScore, it significantly improves detection of csPC, particularly in men considered for biopsy or with PI-RADS 3 lesions [120,121,122]. However, the routine use of TMPRSS2–ERG gene fusion as a biomarker remains limited and is primarily research-oriented.

Extracellular vesicle (EV)-based RNA biomarkers, including the European EPI-CE and FDA-approved ExoDx Prostate (EPI) tests, measure exosomal RNA such as ERG, PCA3, and SPDEF to help reduce unnecessary biopsies in patients with PSA levels of 2–10 ng/mL or those with prior negative biopsies [120,123,124]. Unlike MyProstateScore, EPI testing does not require post-digital rectal examination urine collection. Similarly, the SelectMDx test quantifies urinary mRNA levels of *DLX1*, *HOXC6*, and *KLK3* and integrates these with clinical factors such as PSA, age, and prostate volume to detect high-grade disease (Gleason ≥ 7), improving biopsy decision-making while reducing overdiagnosis [125,126].

Importantly, multiparametric MRI (mpMRI) of the prostate has transformed the biopsy paradigm, often used alongside or after these biomarker tests. For instance, an elevated PHI or 4Kscore may prompt further imaging with mpMRI, whereas a negative MRI and low biomarker score could safely defer biopsy [127,128,129]. Some advanced algorithms, such as the Stockholm3 model, even combine blood biomarkers, genetic single-nucleotide polymorphisms (SNPs), and clinical variables to determine the need for MRI or biopsy, enhancing personalized risk assessment [130,131,132].

Collectively, these urinary biomarkers, when combined with mpMRI, may offer a more precise, less invasive approach to prostate cancer diagnosis and risk stratification. They can help reduce unnecessary biopsies, detect high-grade cancers earlier, and complement serum markers such as PSA, PHI, 4Kscore, and Proclarix, supporting a multimodal diagnostic workflow that balances sensitivity, specificity, and cost-effectiveness [127,133,134]. While broader clinical implementation remains limited by availability, cost, and technical complexity, these approaches represent a significant advance toward more personalized, evidence-based prostate cancer management in the future. An overview of commercially available biomarker tests used prior to prostate biopsy for risk stratification and biopsy decision-making is provided in Table 6, whereas available biomarker assays used after prostate cancer diagnosis, including tools for prognostication, treatment selection, and monitoring in advanced disease, are summarized in Table 7.

## 8. Future Research Directions

Future progress in the field of PCa biomarkers will depend on addressing several key challenges. Prospective, multi-center clinical trials are required to validate emerging epigenetic and liquid biopsy biomarkers within clearly defined clinical contexts, rather than relying on retrospective or heterogeneous cohorts. Demonstrating clinical utility, rather than statistical association, will be crucial for guideline adoption.

There is a growing need for integrative biomarker models that combine molecular data with imaging (mpMRI, PSMA PET), clinical parameters, and longitudinal disease monitoring. Single biomarkers are unlikely to fully capture the biological complexity and temporal evolution of PCa. Therefore, multi-analyte panels and algorithm-based approaches are expected to play an increasing role.

Advances in liquid biopsy technologies offer opportunities for real-time monitoring of clonal evolution, minimal residual disease, and early treatment resistance. Improvements in assay sensitivity, correction for clonal hematopoiesis, and harmonization of pre-analytical workflows will be critical for broader clinical implementation.

Emerging areas such as epigenetic therapeutics, exosome-based diagnostics, and AI-driven biomarker integration represent promising avenues for future research. As costs decrease and technologies mature, these approaches may enable more precise risk stratification and adaptive personalized treatment strategies.

## 9. Conclusions

PCa represents a biologically heterogeneous disease in which traditional biological parameters and PSA are often not sufficient to support optimal clinical decision-making.

Advances in molecular biology and epigenetics have substantially expanded the repertoire of biomarkers capable of capturing tumor heterogeneity, disease aggressiveness, and therapeutic resistance, providing new opportunities for more precise and individualized patient management.

Epigenetic biomarkers, including DNA methylation patterns and non-coding RNA signatures, offer promise due to their early occurrence in tumorigenesis and relative stability across disease stages. Liquid biopsy technologies—encompassing ctDNA, CTCs, and extracellular vesicles—enable minimally invasive, real-time assessment of tumor evolution and resistance mechanisms, addressing key limitations of single-site tissue biopsies. Several assays based on these principles have already reached clinical implementation and guideline acknowledgment, particularly in biopsy triage, post-diagnostic risk stratification, and treatment selection in advanced disease.

The clinical value of these biomarkers is maximized when they are integrated into multimodal diagnostic and therapeutic pathways that combine molecular data with imaging, clinical variables, and longitudinal monitoring. Rather than replacing established tools such as mpMRI or PSA-based assessment, molecular and liquid biopsy biomarkers function as complementary instruments that refine risk stratification and support more informed, adaptive treatment decisions.

Substantial progress has been made, but most emerging biomarkers remain at an early translational stage. Their clinical implementation will require rigorous prospective validation, standardization of analytical workflows, demonstration of clinical utility, and consideration of cost-effectiveness and accessibility.

In summary, the integration of epigenetic and liquid biopsy biomarkers into prostate cancer management represents a critical step toward precision oncology. Continued multidisciplinary research and thoughtful clinical implementation have the potential to transform prostate cancer care by reducing unnecessary interventions, enabling earlier detection of clinically significant disease, and supporting dynamic, biology-driven treatment adaptation.

## Figures and Tables

**Table 1:** Summary of the most promising methylated gene biomarkers.

Methylated Gene	Sample Source/	Use	Routine Clinical Use?	Evidence Status and Main Limitations	References
*GSTP1*	Prostate biopsy tissue (ConfirmMDx); urine DNA after digital rectal examination (research)	Detection of PCa-associated epigenetic alteration; in tissue, contributes to detection of a “field effect” after a negative biopsy to support repeat-biopsy decisions	NCCN: Listed as part of ConfirmMDx as an optional tool for men considering repeat biopsy after a negative result. EAU: Not recommended for routine use; considered investigational/emerging	Highly frequent promoter hypermethylation in PCa; excellent tissue specificity for PCa diagnosis—97%. Urine-based single-gene GSTP1 assays are not standardized and show variable sensitivity depending on collection and assay methodology. ConfirmMDx is also not used routinely due to moderate sensitivity, variability, lack of large prospective trials, regulatory status	[14,15,16,17,18,19]
*APC*	Prostate biopsy tissue (ConfirmMDx)	Used only as part of a multiplex panel to improve risk stratification after a negative biopsy (repeat-biopsy decision support)	NCCN: Included within ConfirmMDx (optional, repeat biopsy context). EAU: No routine recommendation	Validated primarily as a panel component; limited standalone diagnostic value; performance depends on tissue quality and assay thresholds; limited prospective data and the emerging role of MRI-guided diagnostics	[10,18,19,20]
*RASSF1* (*RASSF1A*)	Prostate biopsy tissue (ConfirmMDx); urine (and serum) DNA	Panel-based “field effect” detection after negative biopsy; Investigational non-invasive urine-based detection/triage	NCCN: Included in ConfirmMDx only; not recommended as a standalone urine test. EAU: Investigational only	Frequently methylated in PCa; urine studies show improved sensitivity when combined with other genes, but evidence requires larger-scale studies and remains heterogeneous and method-dependent	[10,18,19,21,22,23,24,25]
*RARB* (*RARB2*)	Urine DNA, serum, tissue/biopsy specimen	Investigational non-invasive detection/triage; some studies show association with higher Gleason score	NCCN: Not guideline-endorsed. EAU: Not recommended for routine clinical use	Multi-gene methylation panels that include RARB often improve detection metrics compared with single-gene assays, but the evidence remains heterogeneous and method-dependent, with variable accuracy and no standardized clinical thresholds established	[14,18,19,24,26,27]

**Table 2:** Summary of key miRNAs identified as biomarkers.

miRNA	Sample Source	Use	Routine Clinical Use?	Evidence Status and Main Limitations	References
miR-145	Tumor tissue, urine	Downregulation in prostate cancer tissue correlates with adverse clinicopathologic features including higher Gleason score, advanced stage, and PSA levels. However, urine exosomal miR-145 correlated with higher Gleason score	No	Clinical utility not standardized, requires prospective validation	[38,39,40,41]
miR-148a	Urine, plasma/serum	Non-invasive PCa detection support; improves PSA performance (especially in men who fall in the intermediate PSA range of 4–10 ng mL^−1^), especially in combination with miR-375	No	Urine handling and normalization required as it may strongly influence results; further validation needed	[30,42]
miR-375	Urine, Blood/plasma, tumor tissue(potentially)	Non-invasive detection support; helps distinguish PCa vs. BPH/healthy patients and improves PSA when combined with other miRNAs, prognostic survival rater marker for late-stage castration-resistant prostate cancer	No	Results heterogeneous across cohorts; systematic reviews highlight variability and a need for more comprehensive studies, although current evidence is promising	[30,43,44,45]
miR-182	Tumor tissue, Blood/plasma,	Consistently upregulated in PCa tissue and higher plasma levels distinguish prostate cancer from BPH. Tissue expression correlates with progression and can improve risk prediction when combined with Gleason score	No	Mainly retrospective (limited) exploratory evidence with cohort variability across tissue and biofluids; absence of standardized assays and prospective validation currently limits clinical translation	[43,46]
miR-1290	Blood	Prognostic marker in advanced PCa, especially mCRPC; survival risk stratification	No	Limited studies available; new broader-stage validation required	[45]
miR-221-5p	Blood,	Potential discrimination of BPH vs. PCa (reported downregulation in PCa)	No	Limited clinical translation; overlap between benign, inflammatory and malignant states reduces specificity	[31,47]
miR-708-3p	Blood	Potential discrimination of BPH vs. PCa	No	Still investigational—requires independent replication and prospective clinical utility studies,	[31]
miR-151-3p	Plasma	Investigational marker of metastatic castration-resistant PC vs. localized PCa biology	No	So far evidence is based on limited retrospective/cohort-based studies; metastatic signatures are hard to standardize and can be confounded by treatment status and tumor burden, no established clinical thresholds till now	[32]
miR-423-3p	Plasma	Investigational marker of metastatic castration-resistant PC vs. treatment-naive PC	No	Same limitations as other metastatic signatures; platform and cohort variability	[32,48]
miR-126	Tumor tissue, urinary exosomes, plasma	Candidate marker in metastatic vs. localized PCa comparisons, potential risk of disease progression (metastasis) signal in biofluids	No	Current results are still preliminary, prospective validation of clinical impact is lacking	[32,49,50]
miR-152	Tumor tissue expression profiling, serum/plasma	Candidate marker in metastatic vs. localized PCa comparisons, marker for reduced biochemical recurrence-free survival	No	Primary results are promising, but larger cohort studies are required, and the added value over other biomarkers and diagnostic approaches remains unclear	[32,51,52]
miR-21	Urine (also plasma)	Detection/prognostic adjunct; part of proposed urine miRNA panels distinguishing PCa vs. controls; prognostic in panels	No	Variability between studies and lack of standardized, clinically validated assay thresholds limit added value over current diagnostic approaches	[32,53]
miR-16	Tumor tissue profiling, blood, urine	Part of multi-miRNA panels for metastasis and prognosis, detectable in blood and urine exosomes where it may correlate with disease stage	No	Clinical role still not clear, clinical assay standardization needed	[32,54,55]
miR-205	Tumor tissue profiling, urine	Diagnostic/prognostic candidate, possibly associated with tumor aggressiveness	No	Inconsistent prognostic utility	[33,56,57]
miR-214	Tumor tissue expression profiling, urine exosomes	Diagnostic/prognostic candidate	No	Remains investigational—noted downregulation in tissue and urinary samples	[33,58]
miR-221	Tumor tissue expression profiling, serum (limited data)	Diagnostic/prognostic candidate for PC, especially associated with metastatic PC	No	Clinical larger-scale validation, standardized assays required	[33,59]
miR-99b	Tumor tissue expression profiling, urine	Diagnostic/prognostic candidate linked with tumor aggressiveness, e.g., for patients after radical prostatectomy	No	Limited data, requires further testing	[33,60,61]
miR-200c	Tumor tissue expression profiling, urine samples	Investigational improvement of Gleason score prediction	No	Needs further reproducible larger-scale assays and proof of benefit beyond MRI/biopsy pathology	[34,62]
miR-200b	Tumor tissue expression profiling	Investigational association with bone metastasis, bilateral disease, PSA > 10 ng/μL, Gleason score	No	Found association which needs further examination to assess its clinical utility	[34]

**Table 3:** Main lncRNAs proposed as biomarkers.

lncRNA	Sample Source	Use	Routine Clinical Use?	Evidence Status and Main Limitations	References
PCA3	Post digital rectal examination (DRE) urine (urinary PCA3 score normalized to PSA mRNA)	Decision support for repeat biopsy after a prior negative biopsy; provides information partly independent of serum PSA	Yes. Progensa PCA3 (FDA-approved urine test; not mentioned in the new Prostate Cancer Early Detection NCCN 1.2026 guidelines)	Moderate sensitivity as a stand-alone marker; requires standardized post-DRE collection; best used as an adjunct, not a replacement for clinical risk models	[64,70,71]
SChLAP1 (SCHLAP1)	Prostate tumor tissue (expression analysis; research/specialized labs)	Prognostic risk stratification: high expression associated with aggressive disease, metastasis, and PCa-specific mortality	No	Still requires assay standardization, validated cut-offs, and prospective studies demonstrating impact on management decisions	[66,72,73]
PCAT family (PCATs as a group)	Mainly prostate tissue; exploratory work in urine/extracellular vesicles	Candidate biomarkers for aggressiveness and prognosis (conceptual lncRNA-based stratification)	No	PCAT refers to multiple transcripts with heterogeneous biology; lack of a locked classifier and prospective validation limits translation	[74,75,76,77]
MALAT1	Primarily tissue; explored in liquid biopsy (urine/plasma) contexts in research	Investigational prognostic/biological marker in PCa and other cancers; potential adjunct to PSA and/or PCA3, potentially associated with tumor growth and invasion	No	Limited disease specificity; context-dependent effects; no defined clinical decision use-case or validated thresholds	[68,78,79,80]
Cuproptosis-related lncRNA panel (e.g., AC005790.1, AC011472.4, AC099791.2, AC144450.1, LIPE-AS1, STPG3-AS1)	Transcriptomic signatures derived from tumor datasets (TCGA-type); limited experimental validation	Proposed diagnostic and prognostic risk-score models (translational research)	No	Requires further validation and proof of added value over established clinical variables	[69]

**Table 4:** Biological and Clinical Distinctions Between ctDNA and CTCs.

Aspect	ctDNA	CTCs
Material availability	Abundant tumor-derived DNA fragments	Rare intact circulating tumor cells
Half-life	Short half-life (minutes–hours)—better for dynamic changes	Longer persistence in circulation
Detection of AR-V7 and other AR resistance mechanisms	Less optimal for splice variants	Better detection (RNA/protein level)
Tumor heterogeneity	Reflects global tumor burden	Reflects viable, aggressive subclones
Phenotypic information	No cellular context	Preserves cell phenotype and morphology
Minimal residual disease (MRD)	High sensitivity for MRD	Lower sensitivity in early disease
Bone metastases detection	Effective regardless of advancement	Often limited due to low CTC shedding
Correlation with WCC/platelet cloaking	No direct correlation	Strong correlation observed
Sensitivity	Very high	Moderate to high (method-dependent)
Specificity	High with targeted assays	High when phenotypically confirmed
Genomic alterations	SNVs, indels, CNVs, methylation	SNVs, CNVs + transcriptomic/proteomic data
RNA analysis	Limited (fragmented RNA)	Robust (full-length RNA, splice variants)
Protein expression	Not assessable	Directly assessable (IHC, IF)
Clonal evolution tracking	Excellent for longitudinal monitoring	Limited by low cell numbers
Pre-analytical stability	Sensitive to handling and timing	Cells relatively stable with proper fixation
Cost and scalability	Lower cost, highly scalable	Higher cost, technically demanding
Standardization	Better standardized across labs	Limited standardization
Clinical implementation	Widely implemented	Mainly specialized/reference centers
Turnaround time	Short	Longer
Source of signal	Apoptotic/necrotic tumor cells	Viable tumor cells
Prognostic value	Strong quantitative prognostic marker	Strong prognostic and predictive marker
Predictive biomarker potential	Mainly genomic	Genomic + phenotypic + functional

**Table 5:** Key technical and translational limitations of epigenetic and liquid biopsy biomarkers in prostate cancer.

Technical Limitation	Biological/Analytical Basis	Clinical Implication	Key References
CTCs require high tumor burden	Extreme rarity of CTCs in peripheral blood and short circulation half-life	Not suitable for population screening or early detection; utility limited to advanced disease	[106,109]
Low ctDNA tumor fraction	ctDNA represents a small proportion of total cfDNA, especially in early-stage disease	Reduced sensitivity for early diagnosis and minimal residual disease	[108,117]
Clonal hematopoiesis (CHIP)	Age-related somatic mutations in hematopoietic cells mimic tumor-derived variants	Risk of false-positive ctDNA findings; need for matched WBC controls	[110,111]
miRNA sensitivity to hemolysis	Release of erythrocyte- and platelet-derived miRNAs during sample handling	Poor reproducibility across studies; strict pre-analytical QC required	[106,112]
Lack of stable miRNA normalization	Reference miRNAs (e.g., miR-16) affected by hemolysis and biological variability	Limited cross-study comparability	[112]
PCA3 normalized to PSA mRNA	Dependence on prostate-derived RNA rather than urine concentration	Variability related to sampling conditions and DRE effectiveness	[118,119]
Tumor heterogeneity	Spatial and clonal diversity of prostate tumors	Single biomarkers insufficient to represent disease biology	[106,117]
Complexity of multimarker panels	Need to integrate genomic, epigenetic, and fragmentomic data	High cost, bioinformatic burden, and limited scalability	[89,108]
Pre-analytical variability and lack of SOPs	Differences in collection tubes, processing time, storage, and transport	Inter-center variability exceeding biological signal	[106,117]
Cautious guideline recommendations	Limited prospective evidence and unclear impact on hard clinical endpoints	No routine recommendation for screening or primary diagnosis	[115,116]

**Table 6:** Commercially available biomarker tests are used before prostate biopsy (risk stratification and biopsy triage).

Test (Company)	Specimen/Matrix	Biomarker Type	Main Clinical Use	Indication (Patient Profile)	Commonly Integrated Tools	Regulatory Status/Availability	Level of Evidence/Guideline Inclusion
PHI (Prostate Health Index) (Beckman Coulter)	Blood	PSA isoform index (tPSA, fPSA, [-2]proPSA)	Improve PSA specificity; biopsy triage	Borderline PSA (commonly 4–10 ng/mL)	PSA density, mpMRI/PI-RADS, nomograms	FDA-approved, CE-marked	Guideline-endorsed (EAU, NCCN, ASCO—optional risk stratification tool)
4Kscore (OPKO Health)	Blood	Four kallikreins + algorithm	Biopsy triage (GG ≥ 2)	Elevated PSA, biopsy consideration	mpMRI/PI-RADS, PSA density, ERSPC/PCPT calculators	LDT (USA), variable EU access	Guideline-endorsed/acknowledged (NCCN, EAU—conditional use)
SelectMDx (MDxHealth)	Urine after DRE	mRNA (HOXC6, DLX1) + clinical data	Biopsy triage (csPCa risk)	PSA 2–10 ng/mL, first biopsy	mpMRI/PI-RADS, PSA density, risk calculators	CE-IVD; LDT (USA)	Guideline-acknowledged (NCCN optional; EAU notes limited added value vs. MRI)
ExoDx Prostate (EPI) (Bio-Techne)	Urine (no DRE)	Exosomal RNA (PCA3, ERG, SPDEF)	Rule-out csPCa; reduce unnecessary biopsies	PSA 2–10 ng/mL, biopsy consideration	mpMRI (PI-RADS 3), PSA density	Commercial LDT (USA)	Guideline-acknowledged (NCCN, AUA; strongest evidence among urine tests)
MiPS/MyProstateScore (MPS) (LynxDx)	Blood + urine after DRE	PSA + PCA3 + TMPRSS2:ERG	Rule-out csPCa	Elevated PSA, equivocal risk	mpMRI, PSA density	Commercial LDT (USA)	Evidence-supported (no formal endorsement)
Progensa PCA3 (Hologic)	Urine after DRE	lncRNA (PCA3)	Support biopsy/repeat biopsy decision	PSA elevation, diagnostic uncertainty	PSA, mpMRI, nomograms	FDA-approved, CE	Guideline-acknowledged (EAU, NCCN—declining role)
miR Sentinel™ (miR Scientific)	Urine	Multi-miRNA profile	Non-invasive detection and risk classification	Initial triage of suspected PCa	PSA, mpMRI, risk calculators	Early commercial stage (USA)	Emerging/investigational
ConfirmMDx (MDxHealth)	FFPE tissue from a negative biopsy	DNA methylation (GSTP1, APC, RASSF1)	Identify occult cancer; re-biopsy decision	Negative biopsy with persistent suspicion	mpMRI, PSA kinetics	CE-IVD; LDT (USA)	Guideline-acknowledged (EAU—limited, mainly pre-MRI era)

**Table 7:** Commercially available biomarker tests are used after diagnosis and in metastatic disease.

Test (Company)	Specimen/Matrix	Biomarker Type	Main Clinical Use	Disease Setting	Commonly Integrated Tools	Regulatory Status/Availability	Level of Evidence/Guideline Inclusion
Oncotype DX GPS (Exact Sciences)	Biopsy/RP tissue	17-gene expression panel	Prognosis, AS vs. treatment	Localized PCa (GG1–GG2)	NCCN risk groups, CAPRA, mpMRI	LDT (USA); global access	Guideline-endorsed (conditional) (NCCN, ASCO)
Prolaris (CCP score) (Myriad)	Biopsy/RP tissue	Cell-cycle gene expression (31 genes)	Progression, mortality risk	Localized PCa	CAPRA/CCR, nomograms	CE; LDT (USA)	Guideline-endorsed (conditional) (NCCN, ASCO, NICE)
Decipher Prostate (Veracyte)	Biopsy/RP tissue	22-gene genomic classifier	Metastasis risk; RT decision-making	Post-RP/selected localized cases	PSA kinetics, MDT, nomograms	LDT (USA)	Guideline-endorsed (NCCN, ASCO, AUA/ASTRO—strongest genomic evidence)
ProMark (Metamark)	Biopsy tissue	Proteomic biomarker panel	Tumor aggressiveness	Localized PCa (GG1–GG2)	mpMRI, NCCN risk groups	Commercial (USA)	Guideline-acknowledged (EAU—limited use)
CellSearch CTC (Menarini)	Blood	CTC count (EpCAM+)	Prognosis, treatment monitoring	Metastatic PCa/mCRPC	PSA kinetics, imaging, labs	FDA-cleared	Guideline-acknowledged (EAU, ASCO—prognostic only)
AR-V7 (Epic Sciences)	Blood (CTCs)	AR-V7 nuclear protein	Predict resistance to ARSI	mCRPC	Imaging, PSA kinetics	LDT (USA)	Guideline-acknowledged (NCCN, ASCO)
AdnaTest AR-V7 (Qiagen)	Blood (CTCs)	AR-V7 mRNA	Resistance prediction	mCRPC	Clinical course, ctDNA	CE/RUO	Evidence-supported (no formal endorsement)
FoundationOne Liquid CDx (Roche)	Blood (ctDNA)	Broad NGS panel	Companion diagnostic for PARP inhibitors	mCRPC	MDT review, tissue confirmation	FDA-approved CDx	Guideline-endorsed (EAU, NCCN, ASCO)
Guardant360 (Guardant Health)	Blood (ctDNA)	NGS panel	Molecular profiling	mCRPC	Molecular tumor board	Commercial	Guideline-acknowledged (commonly used; PCa often off-label)
Tempus xF/Caris MI	Blood (ctDNA)	NGS	Resistance, pathway profiling	Advanced/multiline PCa	MDT interpretation	Commercial	Evidence-supported (no formal endorsement)

## Data Availability

The original contributions presented in this study are included in the article. Further inquiries can be directed to the corresponding author.

## Abbreviations

The following abbreviations are used in this manuscript:

PCa	prostate cancer
BPH	benign prostatic hyperplasia
MiRNA	microRNA
ncRNA	non-coding RNA
lncRNA	long non-coding RNA
circRNA	circular RNA
ctDNA	circulating tumor DNA
cfDNA	cell-free DNA
CTCs	circulating tumor cells
PSA	prostate-specific antigen
PHI	prostate health index
PCA3/DD3	prostate cancer antigen 3
EPI	ExoDx prostate
mCRPC	metastatic castration-resistant prostate cancer
mHSPC	metastatic hormone-sensitive prostate cancer
OS	overall survival
PFS	progression-free survival
WCC	white blood cell count
csPC	clinically significant PC
EV	extracellular vesicle
PSM-E	proteasome subunit alpha type-6
mpMRI	multiparametric MRI
SNPs	single-nucleotide polymorphisms
